# Synergistic regenerative therapy of thin endometrium by human placenta-derived mesenchymal stem cells encapsulated within hyaluronic acid hydrogels

**DOI:** 10.1186/s13287-022-02717-2

**Published:** 2022-02-08

**Authors:** Yifeng Lin, Shunni Dong, Xiaohang Ye, Juan Liu, Jiaqun Li, Yanye Zhang, Mixue Tu, Siwen Wang, Yanyun Ying, Ruixue Chen, Feixia Wang, Feida Ni, Jianpeng Chen, Binyang Du, Dan Zhang

**Affiliations:** 1 Key Laboratory of Women’s Reproductive Health of Zhejiang Province and Department of Reproductive Endocrinology, Women’s Hospital, Zhejiang University School of Medicine, Hangzhou, 310006 Zhejiang China; 2 MOE Key Laboratory of Macromolecular Synthesis and Functionalization, Department of Polymer Science and Engineering, Zhejiang University, Hangzhou, 310027 China; 3 Key Laboratory of Reproductive Genetics (Ministry of Education) and Department of Reproductive Endocrinology, Women’s Hospital, Zhejiang University School of Medicine, Hangzhou, 310006 Zhejiang China

**Keywords:** Human placenta-derived mesenchymal stem cells, Hyaluronic acid hydrogels, Thin endometrium, Endometrial repair, Regeneration mechanisms

## Abstract

**Background:**

Thin endometrium is a primary cause of defective endometrial receptivity, resulting in infertility or recurrent miscarriage. Much effort has been devoted toward regenerating thin endometrium by stem cell-based therapies. The human placenta-derived mesenchymal stem cells (HP-MSCs) are emerging alternative sources of MSCs with various advantages. To maximize their retention inside the uterus, we loaded HP-MSCs with cross-linked hyaluronic acid hydrogel (HA hydrogel) to investigate their therapeutic efficacy and possible underlying mechanisms.

**Methods:**

Ethanol was injected into the mice uterus to establish the endometrium-injured model. The retention time of HP-MSCs and HA hydrogel was detected by in vivo imaging, while the distribution of HP-MSCs was detected by immunofluorescence staining. Functional restoration of the uterus was assessed by testing embryo implantation rates. The endometrial morphological alteration was observed by H&E staining, Masson staining, and immunohistochemistry. In vitro studies were further conducted using EdU, transwell, tube formation, and western blot assays.

**Results:**

Instilled HP-MSCs with HA hydrogel (HP-MSCs-HA) exhibited a prolonged retention time in mouse uteri than normal HP-MSCs. In vivo studies showed that the HP-MSCs-HA could significantly increase the gland number and endometrial thickness (*P* < 0.001, *P* < 0.05), decrease fibrous area (*P* < 0.0001), and promote the proliferation and angiogenesis of endometrial cells (as indicated by Ki67 and VEGF, *P* < 0.05, *P* < 0.05, respectively) in mice injured endometrium. HP-MSCs-HA could also significantly improve the embryo implantation rate (*P* < 0.01) compared with the ethanol group. Further mechanistic study showed the paracrine effects of HP-MSCs. They could not only promote the proliferation and migration of human endometrial stromal cells via the JNK/Erk1/2-Stat3-VEGF pathway but also facilitate the proliferation of glandular cells via Jak2-Stat5 and c-Fos-VEGF pathway. In turn, the increased VEGF in the endometrium promoted the angiogenesis of endothelial cells.

**Conclusion:**

Our study suggested the potential therapeutic effects and the underlying mechanisms of HP-MSCs-HA on treating thin endometrium. HA hydrogel could be a preferable delivery method for HP-MSCs, and the strategy represents a promising therapeutic approach against endometrial injury in clinical settings.

**Graphical abstract:**

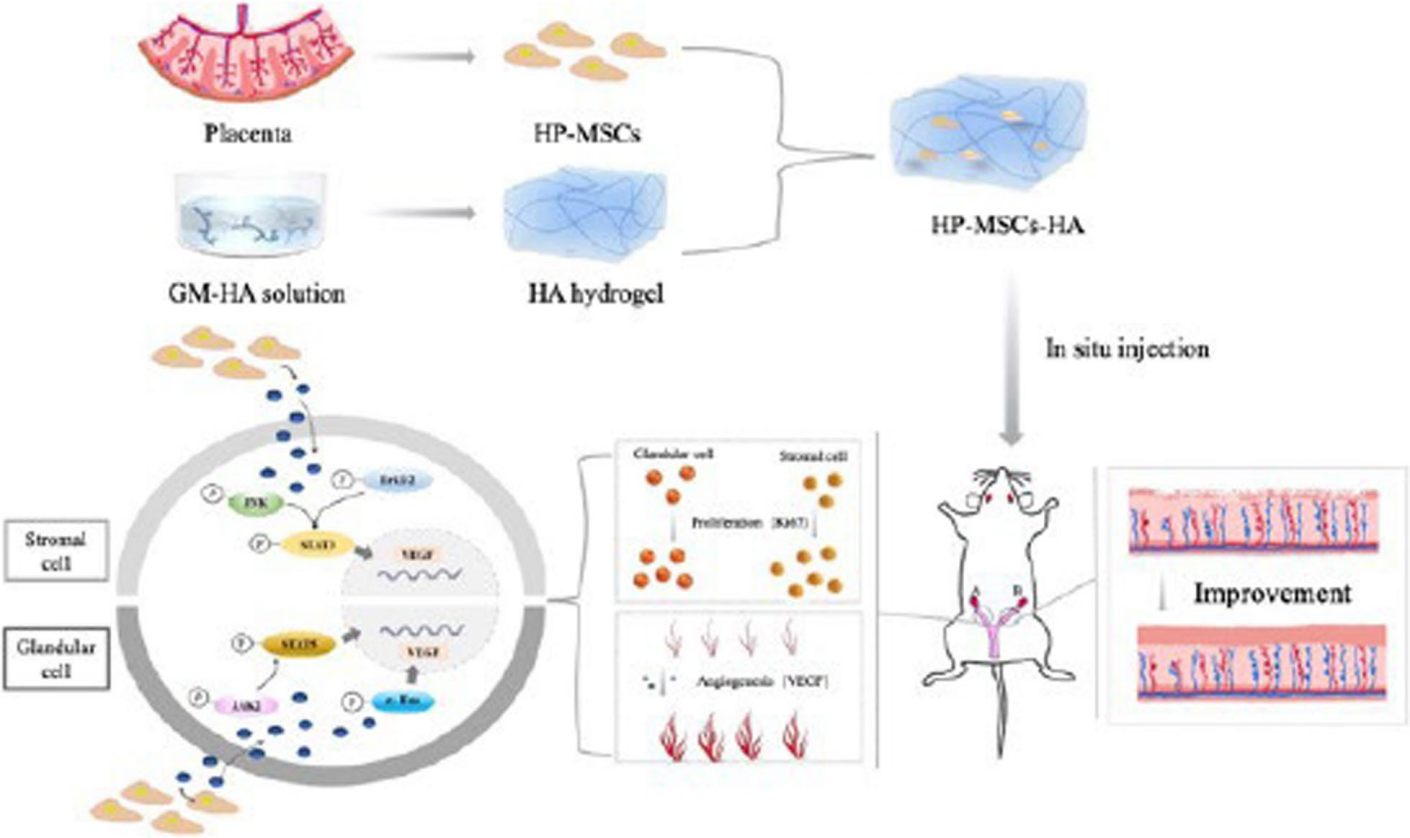

**Supplementary Information:**

The online version contains supplementary material available at 10.1186/s13287-022-02717-2.

## Background

Infertility is estimated to affect 8%-12% of reproductive-aged couples worldwide and is often associated with significant physical and emotional stress [1]. Assisted reproductive technologies (ART) is a great solution for most causes of infertility, such as ovulatory dysfunction, male factor infertility, and tubal disease [2]. However, the implantation rate is still as low as 20–30% due to poor endometrial receptivity [3, 4], among which thin endometrium is a primary cause of failed embryo transfer, resulting in long-term infertility and negative family outcomes. Embryo implantation is first nourished by endometrial gland secretions. However, physical (induced abortion, frequent intrauterine operation, radiation) and biochemical (infection, endocrine-disrupting chemical) injury will impair the normal growth of the endometrium, resulting in thin endometrium, a common disease in women of reproductive age [5, 6]. In assisted reproduction, an endometrial thickness of 8–12 mm is considered normal for embryo implantation, a thickness of less than 7–8 mm is commonly defined as thin endometrium [6–9]. Previous studies have reported a significant correlation between thin endometrium and infertility. The mean endometrial thickness was significantly higher in pregnant women than non-pregnant women [10–13]. Therefore, improving endometrial thickness in patients with thin endometrium will certainly benefit the pregnancy process and achieve positive family outcomes.

Over the years, several treatment modalities have been suggested for thin endometrium, including hysteroscopic adhesiolysis, hormonal manipulation, and adjuvants (aspirin, luteal estradiol, sildenafil citrate, granulocyte colony-stimulating factor) [14]. Despite the vast diversity of treating methods, only a minor facilitating change is reported in the endometrium thickness and implantation rates without robust evidence, especially for severe endometrium-injured patients [14]. Thus, treatment of thin endometrium remains a challenge, and future studies are crucial to improving pregnancy outcomes in patients with thin endometrium.

In the field of cell therapy, stem cell transplantation has pioneered novel approaches for the biomedical treatment of injured endometrium [15, 16]. MSCs are undifferentiated pluripotent stem cells with the characteristics of paracrine, immune regulation, and multi-directional differentiation [17]. The origins of MSCs include autologous bone marrow, autologous peripheral blood, autologous muscle, allogeneic fetal liver, allogeneic umbilical cord, etc. MSCs infusion therapy has been applied in animal models and preclinical experiments of various diseases and has shown favorable outcomes in relieving animal symptoms and improving patients' comprehensive signs [17, 18]. For the therapy of thin endometrium, there are only a handful of reported cases hitherto, among which bone marrow-derived mesenchymal stem cells (BM-MSCs) are principally studied, while the major focus is on the therapeutic results, yet the underlying mechanism is not clear [19]. However, BM-MSCs are difficult to acquire for large-scale uses owing to the invasive process of bone marrow collection, which leads to an increased risk of microbial infection. In contrast, the allogeneic placenta is a bona fide candidate origin of MSCs. Abundant MSCs have been found in the placenta, the process of placenta collecting noninvasive, which causes no harm to the fetus and the mother [20, 21]. Moreover, owing to the low immunogenicity of HP-MSCs, they can be applied to treat animal models and patients without causing immune responses [5–9]. Besides, recent research indicates that HP-MSCs also show vigorous expansion ability and greater proliferation capacity than umbilical cord-derived MSCs (UC-MSCs) [22]. Taken together, all these favorable conditions make HP-MSCs to be a novel alternative source for stem cell therapy in treating thin endometrium.

Physiologically, the uterus is closely interlinked with the vagina. Thus, the traditional in situ instillation of HP-MSCs tends to cause part of the cell suspension to slip out of the uterus via the vagina, limiting the maximal retention efficacy of HP-MSCs. Several novel strategies have been adopted to enhance the retention time of stem cells, including hyaluronic acid (HA) and Pluronic F-127 [15, 23–27]. Among those, HA hydrogel is a promising candidate for the encapsulation and release of HP-MSCs for endometrial therapy. The existence of hyaluronidase in the endometrium can effectively degrade HA in case that HA stays in the uterine cavity persistently without decomposition [28]. Moreover, HA hydrogel has a three-dimensional cross-linked network and shows low interfacial tension and adhesion characteristics, which can provide structural and mechanical support for adjacent cells. As a result, HP-MSCs can be gradually released from the hydrogel to achieve the optimal therapeutic effect [26].

The human endometrium is a complex and dynamic tissue that consists of an outer functionalis layer and an inner basalis layer. The functionalis is mainly composed of glandular cells and stromal cells, while the inner basalis layer contains mainly stromal cells [29]. The two layers are nourished by spiral arteries. Consequently, exploring the effects and underlying mechanisms of HP-MSCs on primary human endometrial cells and human umbilical vein endothelial cells (HUVECs) might help us improve cell therapy for the thin endometrium.

In the present study, we encapsulate the HP-MSCs within a HA hydrogel(HP-MSCs-HA)and then evaluate the therapeutic effects of HP-MSCs-HA on the murine model of thin endometrium in vivo, including the embryo implantation rate, the endometrial thickness, the number of glands, the degree of fibrosis, and the proliferation and angiogenesis. Furthermore, we probe the potential molecular mechanism and signal pathway of HP-MSCs on endometrial stromal and glandular cells in vitro.

## Methods

### Materials

Hyaluronic acid (HA, 97%) and glycidyl methacrylate (GMA, 98%) were purchased from Maclin Biochemical Co. Ltd (Shanghai, China) and J&K Chemical Ltd (Beijing, China)*,* respectively. 2-hydroxy-4’-(2-hydroxyethyl)-2-methylpropiophenone (Photoinitiator Irgacure 2959, 99%) was obtained from Aladdin Bio-Chem Technology Co. Ltd (Shanghai, China). Fluorescein O-methacrylate (FMA, 97%) was acquired from Sigma-Aldrich (Pennsylvania, USA). All chemicals were used as received without further purification.

Identified HP-MSCs were kindly provided by the College of Life Sciences-iCell Biotechnology Regenerative Biomedicine Laboratory, Zhejiang University. The identification results of these HP-MSCs were provided in supplement materials. The cells were cultured in basic DMEM/F12 medium (Hyclone, Logan, UT, USA) containing 10% fetal bovine serum ﻿(FBS, Biological Industries, Kibbutz Beit-Haemek, Israel), 1% penicillin (Geno, Hangzhou, China) at 5% CO_2_ and 37 °C as previously optimized [30, 31]. HUVECs were purchased from ScienCell company (8000, California, USA) and were cultured in ECM medium (1001, ScienCell, California, USA) at 37 °C with 5% CO2.

### Preparation and characterization of HA hydrogel

**Fig. 1:**
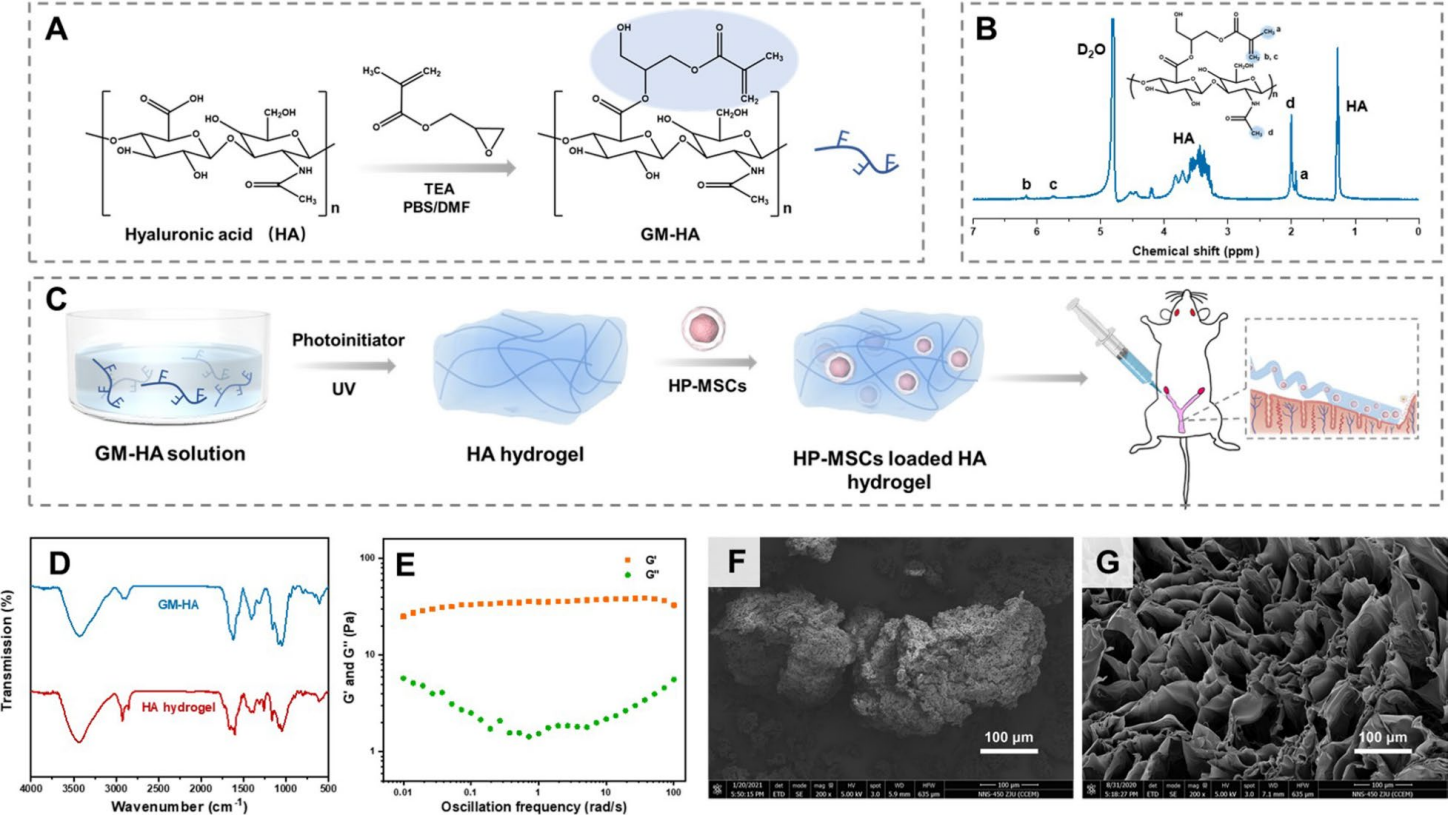
Fabrication and characterization of HA hydrogel. **A** The synthesis routine of GM-HA. **B** The ^1^H-NMR spectrum of GM-HA in D_2_O. **C** Schematic presentation of preparation procedures of HA hydrogel and HP-MSCs-HA as well as the instillation of HP-MSCs-HA into a mouse model. **D** The FT-IR spectra of GM-HA and HA hydrogel. **E** The storage modulus G’ and loss modulus G’’ of HA hydrogel versus oscillation frequency from 0.01 to 100 rad/s with a fixed stain of 0.2% at 37 °C. **F** SEM morphology of freeze-dried HA. **G** SEM morphology of HA hydrogel. The scale bars in (**F**) and (**G**) were 100 μm

The polymerizable HA was prepared via the chemical modification of HA by using GMA, as shown in Fig. 1A. Briefly, 0.5 g HA was dissolved in 100 mL mixture of *N, N*-dimethylformamide (DMF), and phosphate buffer saline (PBS) solution (0.01 M, pH = 7.40) with a volume ratio of 1:1 overnight. The mixture was subsequently mixed with 3.35 g of triethylamine (TEA) for 30 min. Then, 6.65 g GMA was added to the mixture and stirred for 5 days at room temperature. The reaction mixture was precipitated in 20 times excess acetone solution. The precipitates were collected and dried in a vacuum oven. The products were purified by dialysis in deionized water for 3 days and further dried by lyophilization, leading to the final GMA functionalized HA powders, coded as GM-HA. To prepare HA hydrogel, 10 mg GM-HA were dissolved in 1 mL aqueous solution containing 1‰ Irgacure 2959 photoinitiator. The mixed solution was then exposed to 365 nm UV light for 15 min, leading to the formation of HA hydrogels. Similarly, the fluorescent HA hydrogel was synthesized by the polymerization between GM-HA (10 mg) and FMA (1 mg) under exposure to 365 nm UV light for 15 min with the same concentration of photoinitiator, which was applied to observe the retention of HA hydrogel in the endometrium.

^1^H-NMR spectrum of GM-HA was recorded on a 400 MHz Bruker NMR instrument with D_2_O as a solvent. Fourier transform infrared (FT-IR) spectra of GM-HA and HA hydrogel were obtained by a Bruker Vector 22 spectrometer. The rheological measurement of HA hydrogel was carried out on DHR2-2183 rheometer at 37 °C. The morphologies of HA and HA hydrogel were observed by Nova Nano scanning electron microscopy (SEM 450, Thermo FEI). The HA and lyophilized HA hydrogel power were evenly spread on the conductive glue and purged three times with an ear wash ball for SEM samples. The prepared samples were sprayed with platinum for 60 s before SEM observation. After that, 500 µL HA hydrogel was mixed with 4 × 10^6^ HP-MSCs and incubated at 37 °C for 10 min to prepare HP-MSCs-HA, and the mouse was injected with 25 µL HP-MSCs-HA per uterus.

### Isolation and culture of Human stromal and glandular cells

Twenty female patients aged between 24–48 years old with regular menstrual cycles (21–35 days) were recruited at Women’s Hospital of Zhejiang University, China. They haven’t received hormone therapy at least three months before surgery. All the patients underwent hysteroscopy and endometrial biopsy during the infertility examination. Stromal and glandular cells were isolated from the endometrium as previously described [32]. Both groups of cells were cultured in DMEM/F12 medium (Hyclone, Waltham, US) containing 10% FBS and 1% Penicillin–Streptomycin at 37 °C in a humidified environment with 5% CO_2_. After co-culturing with HP-MSCs for 24 h, 48 h, and 72 h, respectively, the cells were used to measure EdU/Transwell/Western blot assay.

### In vivo tracing of CM-DiD/CM-DiR-labeled HP-MSCs

The commercial cell membrane red fluorescent probe CM-DiD/CM-DiR (US Everbright® Inc, Jiangsu, China) was diluted with DMSO (Sigma, Darmstadt, Germany) according to the manufacturer’s instructions to make a final concentration of 1 mM. HP-MSCs at a density of 10^6^ cells/mL were suspended in 5 mL PBS. 10 µL CM-DiD or CM-DiR cell-labeling solution was then added at 37 °C ﻿for post-instillation tracking in utero. After 20 min, the labelled cells were spun at 800 × g for 3 min and followed with washing twice with 5 mL PBS at 1000 × g for 5 min each. The CM-DiD and CM-DiR labelled HP-MSCs were then mixed with as-prepared HA hydrogel, respectively, to form two HP-MSCs-HA with different fluorescent labels. The mouse model was divided into two groups, as shown below, one group was injected with CM-DiR labelled HP-MSCs and HP-MSCs-HA for imaging, using an IVIS Spectrum to detect after transplantation for 1, 3, 7, 14, and 35 days, respectively. The other group was injected with CM-DiD label HP-MSCs and HP-MSCs-HA, which was used for the frozen section. Slides with 6 µm thick (cutting with a CryoStar NX50, Thermo) were observed by an Olympus IX81-FV1000 fluorescence microscope.

CM-DiR labeled group:

- Left uteri: instillation of 25 μL HP-MSCs alone (2 × 10^5^ per/uterus, coded as HP-MSCs)
- Right uteri: instillation of 25 μL HP-MSCs-HA (2 × 10^5^ per/uterus, coded as HP-MSCs-HA)

CM-DiD labeled group:

- Left uteri: instillation of 25 μL HP-MSCs alone (2 × 10^5^ per/uterus, coded as HP-MSCs)
- Right uteri: instillation of 25 μL HP-MSCs-HA (2 × 10^5^ per/uterus, coded as HP-MSCs-HA)

### Transwell migration assay

Human stromal cells or glandular cells were cultured in the upper plate compartment (8-μm, 24-well insert, Corning, NY, USA), while the HP-MSCs were cultured in the lower chamber. They were then co-cultured at 37 °C for 24 h, 48 h, and 72 h, respectively. The medium and cells were then removed from the upper chamber using cotton swabs with 1 × PBS, while the migrated cells on the bottom surface of the membrane were fixed and stained with 0.5% crystal violet (Solarbio, Beijing, China) [33]. Cells from 5 random fields were counted using an inverted microscope (OLYMPUS BX61, Japan) with a magnification of 200x.

### EdU proliferation assay

Human stromal cells or glandular cells were plated in 24-well plates and co-cultured with HP-MSCs in the upper insert (0.4-μm, 24-well insert, Corning, NY, USA). After incubated for 24 h, 48 h, and 72 h, respectively, the transwell inserts loaded with HP-MSCs were removed, and cell proliferation activity was assessed using a commercially available EdU Assay Kit (RIBOBIO, Guangdong, China) according to the protocol provided. Cell proliferation was quantified by the incorporation of EdU into the newly synthesized DNA of replicating cells. The proliferated cells were dyed red, while the nuclei of all cells were dyed blue with DAPI. By counting EdU/DAPI ratio, cell proliferation ability can be assessed.

### Endothelial cell tube formation

Cells were seeded in Matrigel-coated u-slides at a density of 1 × 10^4^ cells per well (Ibidi, Germany) and incubated at 37 °C for 4 h. Images were acquired using an inverted microscope (OLYMPUS BX61, Japan). The results were quantified by counting the number of junctions, total tube length, and total branching length using ImageJ 1.52 k.

### Protein isolation and western blot analysis

Proteins were extracted from the treated human endometrial stromal cells and glandular cells and then lysed by RIPA lysis buffer (Cell Signaling Technology, Boston, MA, USA) as previously described [23]. The protein concentration was detected using a bicinchoninic acid protein assay kit (Thermo Scientific, Waltham, USA). Proteins were denatured in a 5 × SDS-PAGE loading buffer (CWBIO, Beijing, China). Then they were separated on sodium dodecyl sulfate–polyacrylamide gels and subsequently transferred onto nitrocellulose membranes. After being blocked with 5% BSA (Albumin from bovine serum) in PBS, the membranes were incubated with primary and secondary antibodies. The immunoblots were washed with PBST (PBS with 0.1% Tween-20). And then, the membranes were incubated overnight with primary antibodies at 4 °C. After washing, secondary antibodies were added and incubated at room temperature for 1 h in the dark. Finally, the ﻿membranes were probed with an Odyssey CLx (LI-COR, USA). The signal intensity was calculated with ImageStudio (LI-COR, USA). Protein expression was normalized to β-Actin.

Rabbit monoclonal anti-p-JNK (9255), rabbit monoclonal anti-JNK (9252), rabbit monoclonal anti-p-Stat3 (9145), mouse monoclonal anti-Stat3 (9139), rabbit monoclonal anti-p-Erk1/2 (4370), rabbit monoclonal anti-Erk1/2 (4695), rabbit monoclonal anti-p-Jak2 (3771), rabbit monoclonal anti-Jak2 (3230), rabbit monoclonal anti-Stat5 (D3N2B), rabbit monoclonal anti-p–c-Fos (5348), rabbit monoclonal anti-c-Fos (2250), rabbit monoclonal anti-p-c-Jun (3270), and rabbit monoclonal anti-c-Jun (9165) were purchased from Cell Signaling Technology. Mouse monoclonal anti-β-Actin (﻿sc-47778) was purchased from Santa Cruz. Rabbit monoclonal anti-VEGF (GTX102643) was purchased from Gene Tex. Goat anti-mouse fluorescent antibody (926-68020) and Goat anti-rabbit fluorescent antibody (926-32211) were purchased from LI-COR (USA).

### Establishment of the endometrium-injured mouse model

An 8-week-old female ICR mice of clean grade (SLAC company, Shanghai, China) were reared in the animal center under controlled conditions (21–24 °C, relative humidity 40–60%, 12 h light / 12 h dark cycle), with free access to food and water. To mimic endometrial injury in the clinical setting, we established a mouse model of thin endometrium by both mechanical and chemical injury at the estrous period. Briefly, the surgery involves mechanical intrauterine operation with a syringe contacting the uterine cavity and chemical injury by intrauterine perfusion with ethanol (95%) [19, 34, 35]. The mice were randomly assigned into five groups: sham-operated (PBS) group I, of which the uterine cavity was injected with 25 μL PBS and held for 3 min; ethanol group II, for which 25 μL 95% ethanol was injected into the uterine cavity to induce damage for 3 min followed by 25 μL PBS treatment; HA-treated group III, administration of 25 μL HA after endometrium damage by 25 μL 95% ethanol as described above; HP-MSCs-treated group IV, administration of 25 μL HP-MSC (2 × 10^5^ per/uterus) after inducing endometrium damage by 25 μL 95% ethanol; and HP-MSCs-HA-treated group V, administration of 25 μL HP-MSCs-HA (2 × 10^5^ HP-MSCs per/uterus) after inducing endometrium damage by 25 μL 95% ethanol. After acclimation for 7 days, the mice were killed for further analyses. Additional file 1: Fig. S1 showed the construction and treatment procedure of the endometrium-injured mouse model in eight steps, which were briefly described as followings: I: mouse anesthesia; II: shaving the back of mouse; III: disinfecting exposed areas; IV: uteri exposure; V: instilling 25 μL ethanol in the uterine cavity and holding 3 min to fully establish the model of thin endometrium; VI: intrauterine instillation of 25 μL treating materials; VII: muscle suture; and VIII: closure of back skin incision.

### Histological analysis and immunohistochemistry

Standard H&E staining was used for murine endometrial assessment. 30 treated female mice (6 mice, 12 uteri in each group) were euthanized on day 7 after surgery. The isolated uteri were embedded in paraffin after fixing with 4% paraformaldehyde overnight. The wax blocks were cut into 3–4 μm thick and stained with Hematoxylin–Eosin staining by standard methods to observe the endometrial thickness and the number of glands. Light microscopy photographs (OLYMPUS VS200, Japan) and endometrial images were analyzed using the Application Program Image-Pro Plus (version 6.0). Endometrial area and perimeter were recorded to assess an average measurement of the endometrial thickness (mean endometrial thickness = area/perimeter). Tissue sections were also labelled with Masson’s trichrome to measure the degree of endometrial fibrosis by conventional methods. The area of fibrosis was quantified by measuring the area ratio between endometrial stromal fibrosis and the endometrial area using a quantitative image analysis system (Image-Pro Plus software; Media Cybernetics, Bethesda, MD). The immunofluorescence staining was conducted as previously described [32]. Sections were incubated with primary antibodies Ki67, a rabbit polyclonal primary antibody (ab16667; 1:200; Abcam, Cambridge, UK). The secondary antibody (GK600711; DakoCytomation, Glostrup, Denmark) was applied for 30 min at room temperature. The number of positive staining cells in the glands and stroma was semi-quantitatively scored by the immune response score (IRS) of two observers who did not know the source of the samples. The scoring criteria were the same as previously described [32].

### Fertility assessment

Forty treated female mice (8 in each group) were mated with fertile males of the same age (female/male = 1:1) 7 days after surgery. The female mice were checked for vaginal plugs the next morning to determine whether pregnancy had occurred. On the seventh day after the initial detection of vaginal plugs, the mice were sacrificed and the location and number of embryo implantation were recorded by photography.

### Statistical analysis

The collected data were analyzed using GraphPad Prism, ver. 8.4.0 software. All results were presented as mean ± SEM. The software compared the means of samples using student's t-test between two groups and one-way analysis of variance (ANOVA) test among multiple groups. P-value < 0.05 was considered statistically significant.

## Results

### Fabrication and characterization of HA hydrogel

As shown in Fig. 1A, the polymerizable GM-HA was prepared by the ring-opening reaction between HA and glycidyl methacrylate in PBS/DMF solution. Figure 1B shows the ^1^H-NMR spectrum of GM-HA with D_2_O as the solvent, of which the peaks of ~ 5.72 and ~ 6.16 ppm can be assigned to the vinyl protons of methacrylate. Besides, the peaks of ~ 1.93 and ~ 2.00 ppm represented the methyl protons of methacrylate and acetamide in GM-HA, respectively. The degree of methacrylation in GM-HA was estimated to be about 29% according to the ratio of integrated intensity between methacrylate protons and methyl protons in GM-HA acetamide. Figure 1C demonstrates the fabrication and instillation process of HP-MSCs-HA for endometrial therapy. The injectable HA hydrogel was prepared by the photocrosslinking of 1% GM-HA aqueous solution with 1‰ photoinitiator under UV (365 nm) irradiation for 15 min. The HP-MSCs were then encapsulated by HA hydrogel by uniformly mixing HP-MSCs with HA hydrogels, leading to the formation of HP-MSCs-HA. Figure 1D shows the FT-IR spectra of GM-HA and HA hydrogel. The absorption peak at 1630 cm^−1^ was attributed to the carbon–carbon double bond of methacrylate group. For HA hydrogel, a new absorption peak at 1250 cm^−1^ appeared, which can be assigned to the ether group next to the benzene ring of Irgacure 2959. After cross-linking reaction of the carbon–carbon double bond, the absorption intensity at 1630 cm^−1^ clearly decreased. The characteristic peaks of HA mainly contained the stretching vibrations of pyranose ring, glycosidic linkage, amide, ester, -OH, and -NH groups. Among them, the peaks near 2900 cm^−1^ and 1400 cm^−1^ belonged to the stretching vibrations of C-H and bending vibrations of -CH_2_, respectively. The peaks of the pyranose ring and glycosidic linkage can be observed around 1050 cm ^−1^. The broad peak between 3250 to 3500 cm^−1^ was the typical O–H and N–H stretching vibrations. The peaks at 1550 cm ^−1^ to 1700 cm^−1^ can be attributed to the amide and ester groups. The rheology measurements were performed to verify the formation of hydrogel. Figure 1E describes the storage modulus (G’) and loss modulus (G’’) of HA hydrogel as a function of oscillation frequency at 37 °C, which reflected the elasticity and viscosity of the hydrogel, respectively. It is shown that G’ of HA hydrogel was larger than the corresponding G’’ in the oscillation frequency range of 0.01 to 100 rad/s, indicating that the elasticity of HA hydrogel dominates over its viscosity, which can guarantee the retention and coverage of HA hydrogel in the uterine cavity. The morphologies of HA and HA hydrogel were further observed by SEM. Figure 1F indicates that HA did not have a homogeneous structure. However, the cross-linked HA hydrogel exhibited a porous and unbroken skeleton, as shown in Fig. 1G, which can thus facilitate the encapsulation and transportation of small molecules or cells.

### The retention time of HP-MSCs-HA in the endometrium

**Fig. 2:**
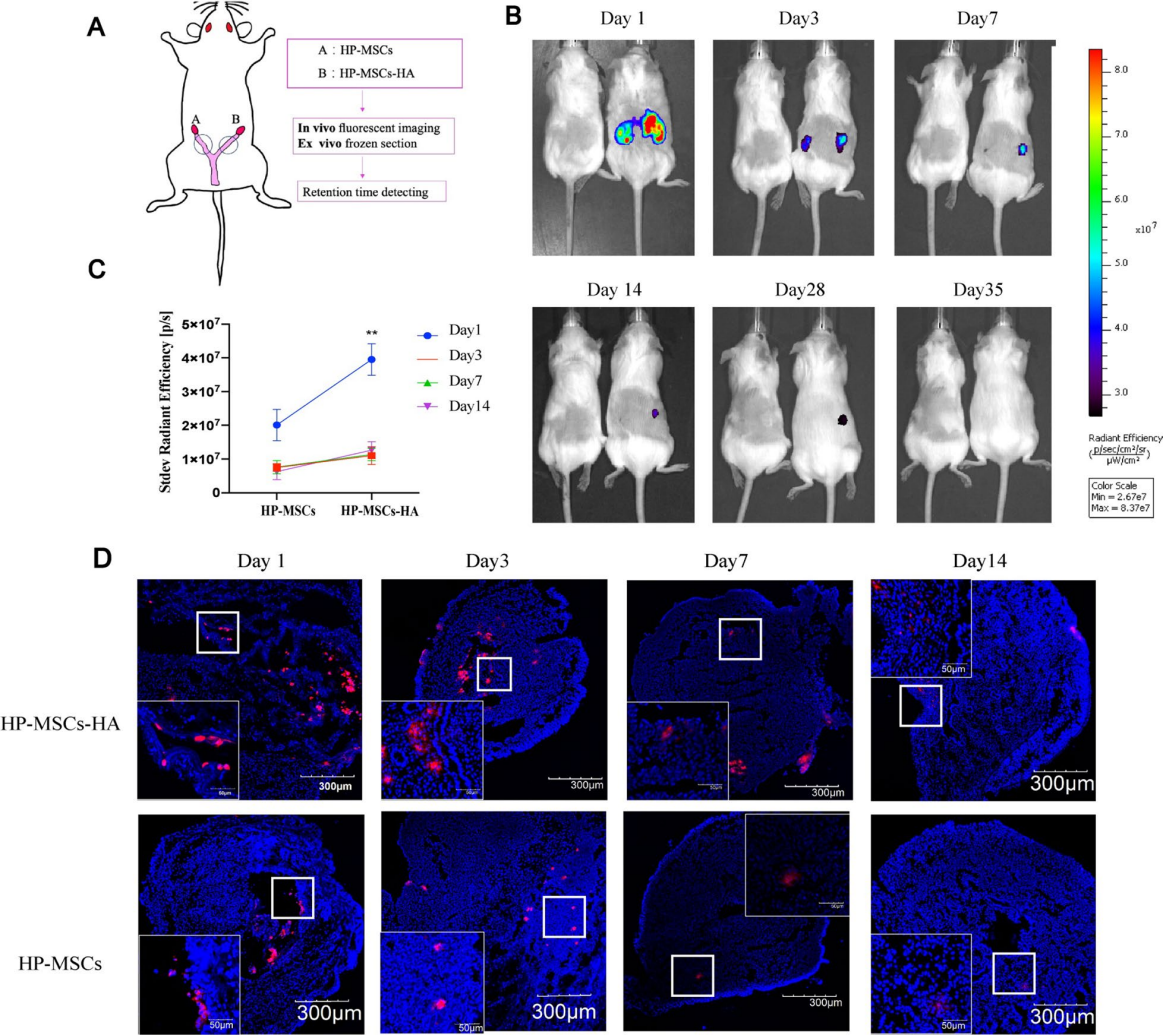
The retention time and distribution of HP-MSCs in the endometrium. **A** Schematic presentation of intrauterine instillation and its corresponding detection methods. Equal cell quantities of HP-MSCs and HP-MSCs-HA labelled by CM-DiD or CM-DiR were implanted to the certain uterus (2 × 10^5^ per/uterus). **B** The mouse on the left was the untreated control group to eliminate the interference of autofluorescence, while the mouse on the right was the treatment group. The retention time of HP-MSCs was observed by an IVIS Spectrum Imaging System at the different time points (1, 3, 7, 14, 28, and 35 days) after transplantation. **C** The total radiant efficiency of the treated uterus was measured and calculated. Data were presented as mean ± SEM (n = 3). ** indicates *P* < 0.01. **D** Ex vivo frozen section of the uterus showed the retention of HP-MSCs, which were labelled with CM-DiD (red). The nuclei of cells were labelled with DAPI (blue)

To achieve optimization of therapeutic efficacy, HP-MSCs-HA was fabricated in the present work to promote the enrichment and maximization of the retention of HP-MSCs in the uterus. First, we studied the kinetics of the HP-MSCs being released from the cross-linked HA hydrogel as well as the retention time of HA hydrogel in murine uterine cavities. Unlike humans with solely one inverted pear shape uterus, there are two tubular uteri of mice, closely interlinked with the vagina. The endometrium-injured mouse model was constructed as described in the experimental section. Then, as shown in Fig. 2A, surgical instillation to the uterine cavity was adopted to provide stem cell in situ therapy. The left uteri of the endometrium-injured mouse model were then given with instillation of HP-MSCs, whereas the right uteri were injected with HP-MSCs-HA. The HP-MSCs were labeled with two fluorescent-labelled fuels, CM-DiD and CM-DiR, respectively. CM-DiD labeling (ex/em: 644/663) was used to track HP-MSCs by ex vivo frozen section, while CM-DiR labeling (ex/em: 748 /780) was suitable for detecting HP-MSCs by *in viv*o imaging. The *in situ* retention of CM-DiR labelled HP-MSCs was observed by an IVIS Spectrum Imaging System at different time points (1, 3, 7, 14, 28, and 35 days, respectively) after transplantation. Figure 2B, C illustrates HA hydrogel prolonged the retention time of HP-MSCs at every checkpoint and showed significant differences on day 1 (20,120,000 ± 4,640,589 p/s vs. 39,525,000 ± 468,817 p/s, *P* < 0.01). Besides, on day 7, the left uterus had few CM-DiR labelled HP-MSCs, while the right uterus injected with HP-MSCs-HA still had prominent HP-MSCs retention, which indicated that the HP-MSCs could stay in the uterus of sexually mature mice for about two estrus cycles. An ex vivo frozen section of the uteri was conducted to confirm this further. As shown in Fig. 2D, the number of HP-MSCs decreased less slowly and exhibited better extension in the HP-MSCs-HA-treated group, consistent with the in vivo imaging results. Besides, these results also indicated that HP-MSCs could infiltrate and distribute in the functional layer and basal layer of the endometrium, which was beneficial to the regeneration and repair of the endometrium.

## In vivo therapy of endometrium-injured mouse model with HP-MSCs-HA

The in vivo endometrial repair effect of HP-MSCs-HA was thus investigated on the endometrium-injured mouse model established by ethanol perfusion to mimic endometrial injury in real clinical infertile patients. The in vivo embryo implantation rate and histological analysis of endometrium, including thickness and gland number, degree of fibrosis, and proliferation effect, were systematically studied.

**Fig. 3:**
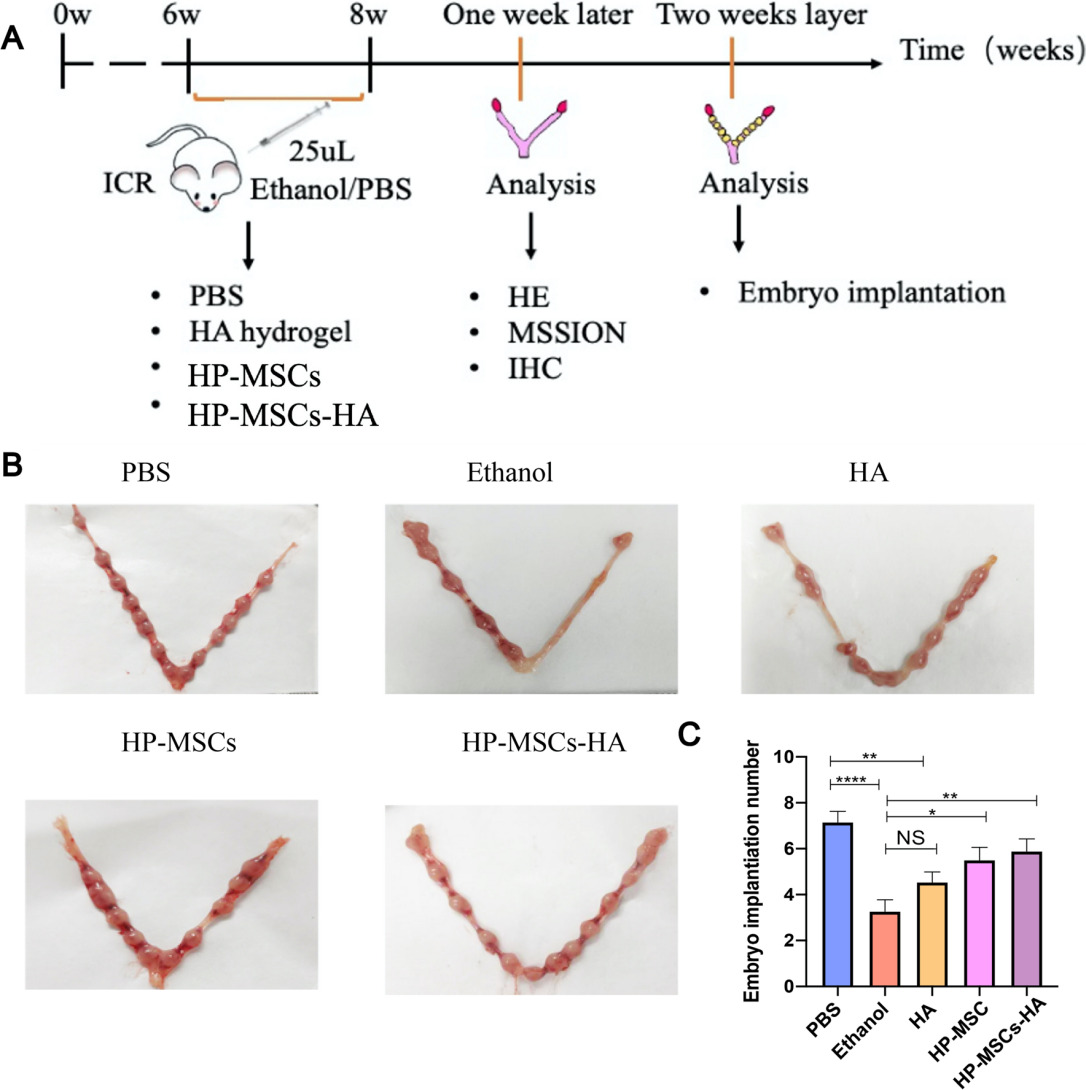
The construction of endometrium-injured mouse model and the evaluation of embryo implantation after therapy. **A** Schematic diagram of mice grouping and their detection methods. **B**, **C** Evaluate the endometrial receptivity of the five mouse groups with different treatments by the number of implanted embryos. * indicates *P* < 0.05, ** indicates *P* < 0.01, **** indicates *P* < 0.0001, n = 8

Embryo implantation refers to the process of early embryo attachment and invasion of the endometrium. In humans, this process begins at the end of the first week after fertilization and endures until the second week of embryo development [36]. Successful embryo implantation requires a receptive endometrium, which allows the uterus to support the development of the embryo and fetus. Therefore, the status of the endometrium can be estimated by measuring the implantation rate of mouse embryos. As shown in Fig. 3A, the 6–8-week-old ICR mice were treated with in situ instillation of 25 µL of 90% ethanol into the uterine to establish the endometrium-injured model. Correspondingly, a control mouse group injected with the same amount (25 µL) of PBS was also set, which was marked as PBS, as shown in Fig. 3B. The endometrium-injured mice were then divided into four groups randomly and were subjected, respectively, to PBS, HA hydrogel, HP-MSCs, and HP-MSCs-HA injection into the injured uterine. The mice groups treated with PBS, HA hydrogel, HP-MSCs, and HP-MSCs-HA were coded as Ethanol, HA, HP-MSCs, and HP-MSCs-HA, respectively (Fig. 3B). One week after surgery, some mice were mated with normal male mice to observe the embryo implantation rate. Figure 3B, C shows that the ethanol group exhibited a remarkable reduction in the number of implanted embryos compared to the PBS group. The average number of fetuses developed in the ethanol group was 3.273 ± 0.506, which is approximately half of the PBS group (7.154 ± 0.478), confirming the successful establishment of the endometrium-injured model. The HA group (4.538 ± 0.447) did not show the prominent difference in the number of fetuses as compared with that of the ethanol group, while the HP-MSC (4.917 ± 0.621) and HP-MSCs-HA group (5.889 ± 0.539) developed a significantly higher number of fetuses as compared with that of the ethanol group. Both the HP-MSCs group and HP-MSCs-HA group demonstrated significantly higher implantation rates compared to the ethanol group.

**Fig. 4:**
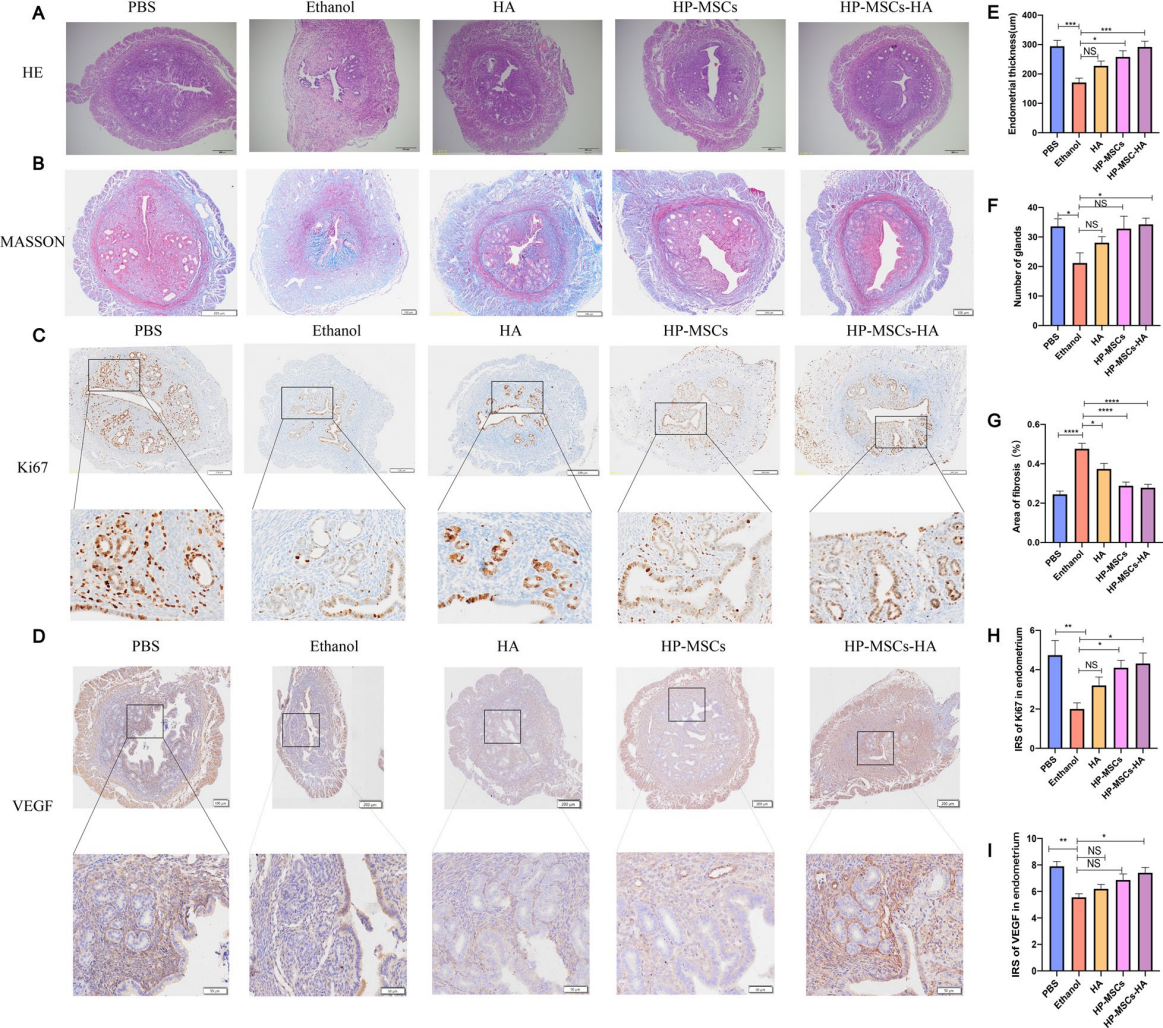
The histological analysis of endometrium for the five mouse groups after different treatments. **A** H&E staining results of the five groups for evaluating the endometrial thickness and number of glands. **B** Masson staining results of the five groups for evaluating the fibrosis status of the endometrium. **C** Immunohistochemical Ki67 expression of the five groups for evaluating the proliferation of endometrial cells. **D** Immunohistochemical VEGF expression of the five groups for evaluating the angiogenesis of endometrium. **E** Average endometrial thickness and statistical analysis (± SEM) of the five groups. * indicates *P* < 0.05, ** indicates *P* < 0.01, *** indicates *P* < 0.001, n = 6. **F** Average gland number and statistical analysis (± SEM) of the five groups. * indicates *P* < 0.05, n = 6. **G** Average fibrosis area and statistical analysis (± SEM) of the five groups. The ratio of the fibrotic area = endometrial fibrotic area/endometrial area. * indicates *P* < 0.05, ** indicates *P* < 0.01, *** indicates *P* < 0.001, **** indicates *P* < 0.0001, n = 6. **H** Statistic analysis of IRS of Ki67 in the endometrium of the five groups. * indicates *P* < 0.05, ** indicates *P* < 0.01, n = 6. **I** Statistic analysis of IRS of VEGF in the endometrium of the five groups. * indicates *P* < 0.05, ** indicates *P* < 0.01, n = 6

Maintaining sufficient endometrial thickness is a precondition for endometrial receptivity and implantation. Patients with thin endometrium have fewer glandular cells than the normal thickness group [6]. H&E staining was applied to evaluate the endometrium thickness as well as the gland number in each group of mice one week after surgery. As shown in Fig. 4A, the ethanol group showed thinner endometrial thickness and fewer endometrial glandular cells than those of the PBS group. After treatment, the most effective endometrial regeneration was observed in the HP-MSCs-HA group, in which their endometrium presented a well-organized structure with epithelia and secretory glands. Besides, the thickness of the endometrial layers in the HP-MSCs-HA group was up to 292.3 ± 19.14 μm, which was approximately twice as thick as that of the ethanol group (171.3 ± 14.59 μm) (Fig. 4E). As shown in Fig. 4F, the number of glandular cells was also significantly improved by HP-MSCs-HA treatment.

One of the commonly described causes of thin endometrium is Asherman syndrome (intrauterine adhesions), which is characterized by injury to the basal layer of the endometrium, leading to endometrial fibrosis [6, 37, 38]. Masson staining that can analyze the fibrous area and the collagen were used to evaluate intrauterine adhesions. The images in Fig. 4B, G revealed that the ethanol group presented higher fibrous tissue (blue) contents of 0.477 ± 0.027 than those of PBS group (0.245 ± 0.017), HA group (0.374 ± 0.027), HP-MSCs group (0.289 ± 0.019), and HP-MSCs-HA groups (0.279 ± 0.016). The HP-MSCs and HP-MSCs-HA group exhibited significant therapeutic outcomes, in which the fibrosis area was not significantly different from that of the PBS group. Additionally, HA hydrogel alone can also restore fibrosis to some extent but is less effective than stem cell treatment.

The proliferation of endometrial cells can be assessed by immunohistochemical staining of Ki67, which can label the cells in the cell cycle. Figure 4C shows the proliferation of endometrial cells and stromal cells by the immunohistochemical staining of Ki67 for the five groups. As shown in Fig. 4H, the IRS scores of ki67 in PBS, ethanol, HA, HP-MSCs and HP-MSCs-HA groups were 4.743 ± 0.745, 2.008 ± 0.306, 3.201 ± 0.427, 4.104 ± 0.369, and 4.319 ± 0.527, respectively. The IRS of Ki67 in the ethanol group was significantly lower than that of the PBS group. Whereas, the HP-MSCs and HP-MSCs-HA group presented an expression of Ki67 close to that of the PBS group.

Angiogenesis is necessary for the regeneration of endometrium and plays a crucial role in developing endometrial receptivity [39, 40]. Miwa et al*.* suggested that reduced endometrial blood flow can result in poor endometrial growth, thus causing thin endometrium [41]. Such a phenomenon can be revealed by the decreased expression of endometrial vascular endothelial growth factor (VEGF): a critical factor in the regulation of endometrial angiogenesis [42]. As shown in Fig. 4D, I, the IRS scores of VEGF in PBS, Ethanol, HA, HP-MSCs and HP-MSCs-HA groups are 7.909 ± 0.3556, 5.571 ± 0.2542, 6.208 ± 0.3225, 6.875 ± 0.4443, and 7.417 ± 0.3981, respectively. The IRS of VGEF in the ethanol group was significantly lower than that of the PBS group. Intriguingly, HP-MSCs-HA treatment could almost rescue VEGF expression, which indicated that HP-MSCs-HA treatment could facilitate angiogenesis in injured endometrium.

These in vivo experimental results indicated the safety and effectiveness of HP-MSCs-HA used for treating thin endometrium in the mouse model.

### Effects of HP-MSCs on the proliferation and migration of human endometrial stromal cell

**Fig. 5:**
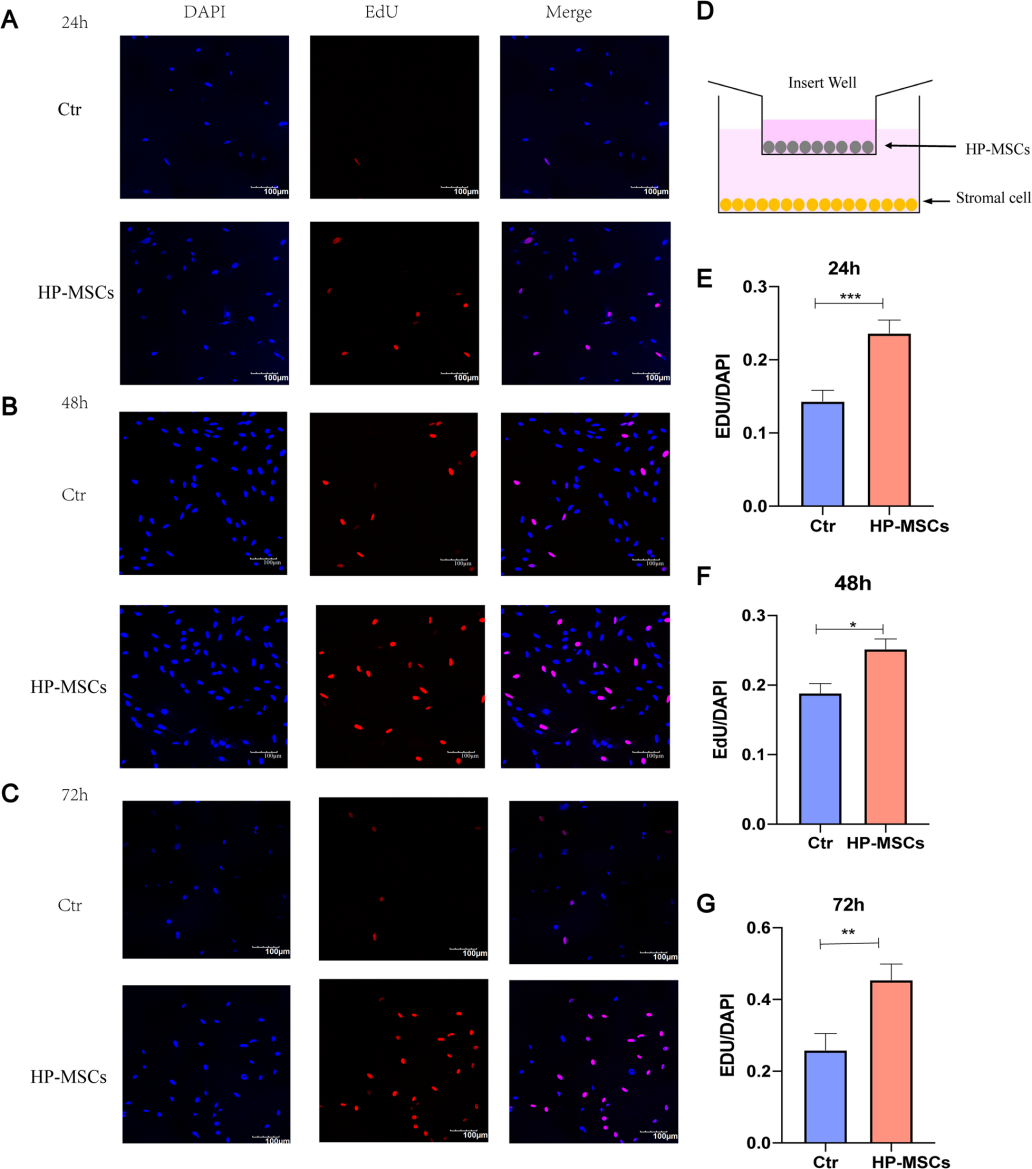
Effects of HP-MSCs on the proliferation of human endometrial stromal cells. EdU assays were conducted at 24 h, 48 h, and 72 h, respectively, after co-culturing of human stromal cells with (Ctr) and with HP-MSCs, respectively. **A**–**C** Representative confocal images of human stromal cells stained with EdU (red) and DAPI (blue) at 24 h, 48 h, and 72 h, respectively. EdU represents the positive proliferation cells, while DAPI represents the cell nucleus. **D** Schematic diagram of co-culture, HP-MSCs were inoculated in the upper insert transwell (0.4um), while stromal cells were in the lower hole plate. **E**–**G** EdU/DAPI represented the ratio of proliferating cells to total cells, and the data were shown as mean ± SEM. * indicates *P* < 0.05, ** indicates *P* < 0.01, *** indicates *P* < 0.001

Based on the therapeutic results, further studies remain to uncover the underlying molecular mechanism. The stromal and glandular cells were thus isolated from the human endometrium to investigate the bioactive effects of HP-MSCs on them in vitro*,* as described in the experimental section. The human primary endometrial stromal and glandular cells were identified by immunohistochemical staining of CK7 and Vimentin, as shown in Additional file 1: Fig. S2. Besides, Fig. 5 illustrates the proliferation effect of HP-MSCs on human stromal cells. The human endometrial stromal cells were cultured without or with HP-MSCs for 24 h, 48 h, and 72 h, respectively, before EdU exposure. EdU/DAPI was the ratio of proliferating cells to total cells that can be used to evaluate cell proliferation. The proliferation rates of stromal cells without and with the presence of HP-MSCs were 14.34 ± 1.492% vs. 23.64 ± 1.795% at 24 h, 18.85 ± 1.358% vs. 25.18 ± 1.456% at 48 h, and 25.86 ± 4.657% vs. 45.5 ± 4.442% at 72 h, respectively (Fig. 5A–C, E–G), confirming that the HP-MSCs significantly enhanced the proliferation ability of stromal cells.

**Fig. 6:**
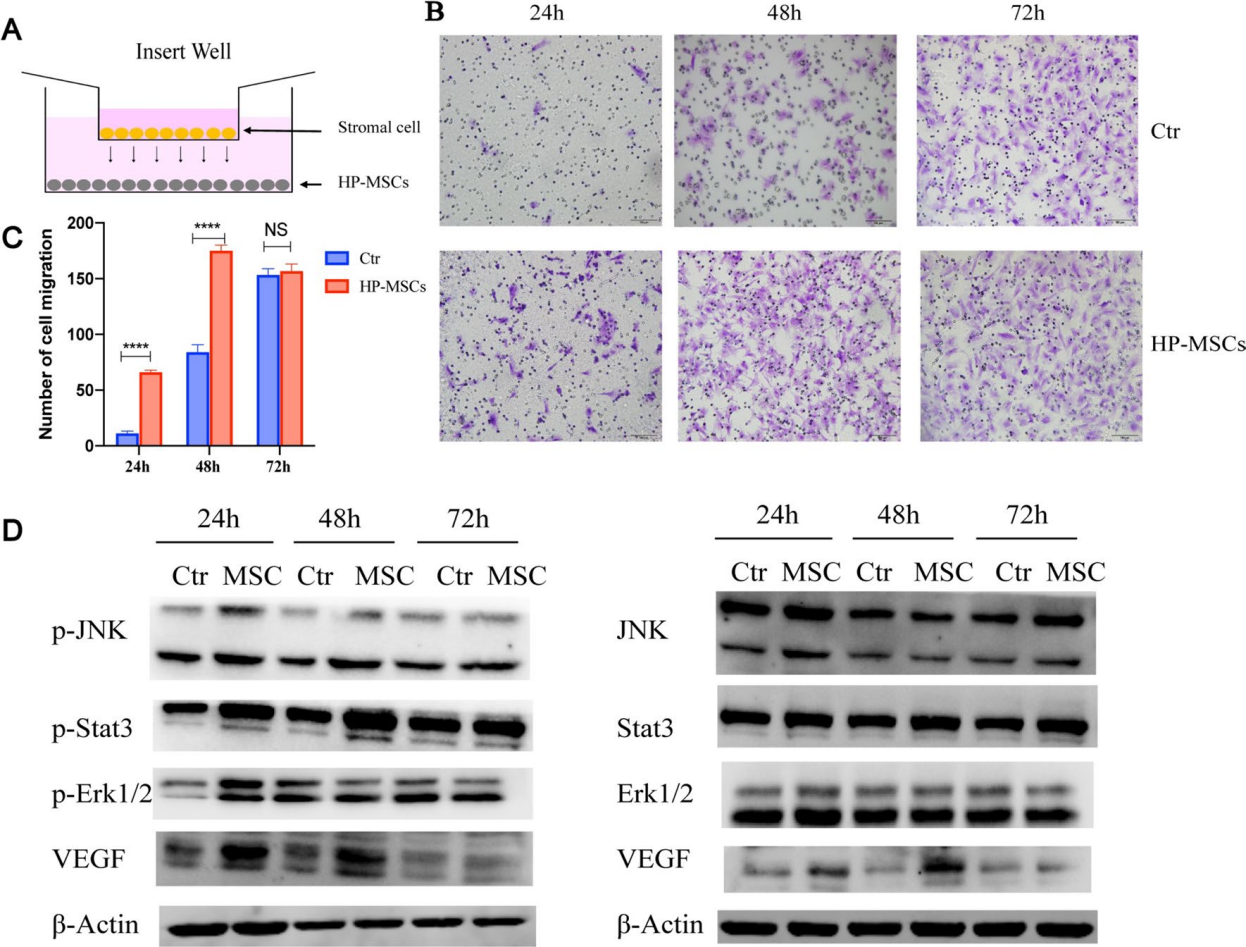
Effect of HP-MSCs on the migration of human endometrial stromal cells. **A** Schematic of co-culturing the stromal cells with HP-MSCs. Stromal cells were inoculated in the upper insert transwell (8 um), while HP-MSCs were in the lower hole plate. **B** Transwell migration assay was conducted at 24 h, 48 h, and 72 h, respectively, after culturing without and with HP-MSCs, respectively. The migrated cells were stained with purple. **C** The average number of migrated stromal cells after culturing without or with HP-MSCs for 24 h, 48 h, and 72 h, respectively (± SEM). * indicates *P* < 0.05, ** indicates *P* < 0.01, *** indicates *P* < 0.001. **D** p-JNK, p-Stat3, p-Erk1/2, VEGF, and corresponding total protein western blot analysis at 24 h, 48 h, and 72 h, respectively, after culturing without and with HP-MSCs, respectively

The effect of HP-MSCs on the migration of human endometrial stromal cells was also conducted. The stromal cells were cultured without (Ctr group) and with HP-MSCs (HP-MSCs group) for 24 h, 48 h, and 72 h, respectively, which were then analyzed by a transwell assay, as shown in Fig. 6A. The numbers of the migrated stromal cells in the HP-MSCs co-cultured group at 24 h and 48 h significantly exceeded that in the corresponding control group (Ctr) without HP-MSCs, as shown in Fig. 6B. However, at 72 h, a minor difference in the number of migrated stromal cells can be observed between the Ctr and HP-MSCs groups. With quantification, the migration rate of stromal cells of the Ctr and HP-MSCs groups were significant as 11.6 ± 1.691% vs. 66.4 ± 1.435% at 24 h and 84.3 ± 6.391% ﻿vs. 175.3 ± 4.807% at 48 h (Fig. 6C), indicating that HP-MSCs not only benefited the proliferation of human endometrial stromal cells but also promoted their migration. To study the underlying mechanisms, we further determine which signaling pathway is involved in regulating the proliferation and migration of stromal cells. Figure 6D shows that co-culturing with HP-MSCs can significantly promote VEGF expression and the phosphorylation level of JNK, Erk1/2, and Stat3 at 24 h in stromal cells. Besides, elevated expression of p-JNK, p-Stat3, and VEGF could be observed until 48 h compared to the control group. Hence with the above results, it can be concluded that co-culturing with HP-MSCs exhibited beneficial effects on the proliferation and migration of human endometrial stromal cells via the JNK/Erk1/2-Stat3-VEGF pathway in vitro.

### Effect of HP-MSCs on the proliferation of human endometrial glandular cell

**Fig. 7:**
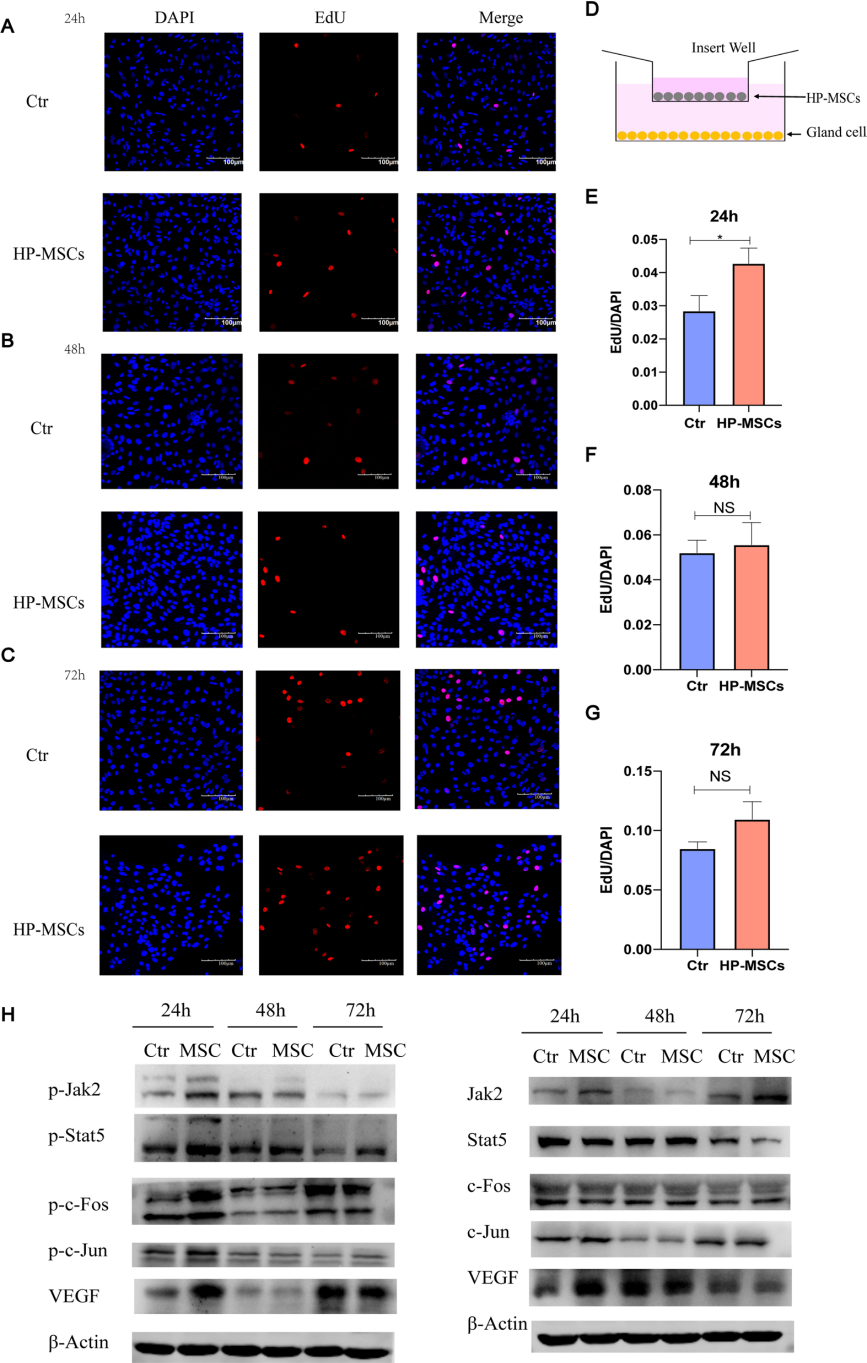
Effect of HP-MSCs on the proliferation of human endometrial glandular cells. **A**–**C** EdU assays were conducted after co-culturing the glandular cells without and with HP-MSCs for 24 h, 48 h, 72 h, respectively. Representative confocal images of the glandular cells stained with EdU (red) and DAPI (blue). **D** Schematic of co-culturing the glandular cells with HP-MSCs. HP-MSCs were inoculated in the upper insert transwell (0.4um), while the glandular cells were in the bottom plate. **E**–**G** Average EdU/DAPI ratio (± SEM). * indicates *P* < 0.05. **H** p-Jak2, p-Stat5, p–c-Fos, p–c-Jun, VEGF, and corresponding total protein western blot analysis at 24 h, 48 h, and 72 h, after culturing without and with HP-MSCs, respectively

In addition to stromal cells, endometrial glandular cells are the other essential component of the endometrium. Similarly, HP-MSCs were co-cultured with the glandular cells for 24 h, 48 h, and 72 h, respectively, and then examined by EdU assay (Fig. 7D). As shown in Fig. 7A–C, the proliferation rates of the HP-MSCs group and the basal medium group at 24 h, 48 h, and 72 h were 2.842 ± 0.463% vs. 4.278 ± 0.463%, 5.203 ± 0.559% vs. 5.561 ± 0.988%, 8.485 ± 0.562% vs. 10.94 ± 1.488%, respectively, which indicated that HP-MSCs could promote the proliferation of human endometrial glandular cells at 24 h (Fig. 7E). However, no significant differences were observed at 48 h and 72 h (Fig. 7F, G).

We further investigated the potential mechanisms of such proliferation enhancement via western blot. As shown in Fig. 7H, the results of western blot validated that HP-MSCs can activate the phosphorylation of c-Fos and upregulate the expression of VEGF protein at 24 h. Besides, elevated expression of p-Jak2 and p-Stat5 were also observed after exposure to HP-MSCs for 24 h. Taken together, these results proved that HP-MSCs co-culturing could improve the migration of human endometrial glandular cells via the Jak2-Stat5 and c-Fos-VEGF pathway, providing favorable conditions for endometrial repair.

### Effect of HP-MSCs on the angiogenesis of HUVECs

**Fig. 8:**
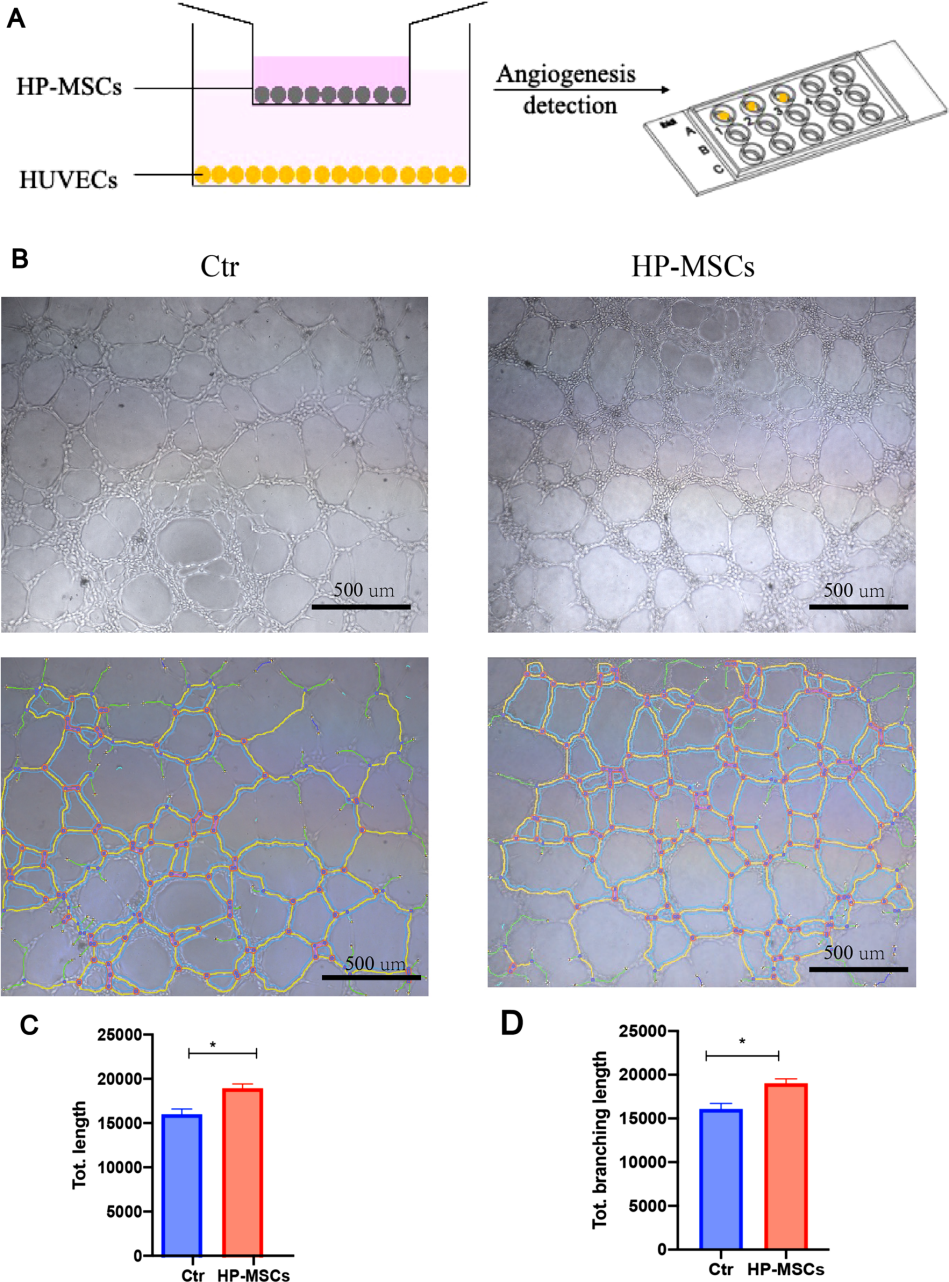
HUVECs angiogenesis assay. **A** Schematic of co-culturing the HP-MSCs with HUVECs. After 24 h co-culturing with HP-MSCs, HUVECs were seeded in Matrigel-coated u-slides to conduct tube formation assay. **B** Representative tube formation images of Ctr and HP-MSCs group. **C**, **D** Quantitative assay of tube formation assay and data were expressed as mean ± SEM. * indicates *P* < 0.05

Previous in vivo studies had revealed the positive effects of HP-MSCs on angiogenesis by increasing the expression of VEGF. To further support the findings of increased VEGF expression that promote angiogenesis, we did the tube formation assay using human umbilical vein endothelial cells (HUVECs). After co-culturing with HP-MSCs for 24 h, HUVECs were seeded in Matrigel-coated u-slides at a density of 1 × 10^4^ cells per well to test their angiogenic ability. As shown in Fig. 8, HP-MSCs co-culturing stimulated significant angiogenesis based on the length and branching length compared with the control (Ctr) group (16,192 ± 602.3 vs. 19,125 ± 507.5, 16,064 ± 646.7 vs. 19,005 ± 529.9, respectively). This result further supported that HP-MSCs-HA treatment could promote angiogenesis in the endometrium.

## Discussion

In this study, we demonstrated a novel strategy to improve the thickness of thin endometrium by HP-MSCs cross-linked with HA hydrogel. HP-MSCs-HA could retain in the endometrium to promote proliferation, migration of stromal cells and glandular cells, as well as angiogenesis, thereby improving embryo implantation rates.

Recent studies have shown that MSCs could serve as a therapeutic agent for thin endometrium caused by endometrial injury [15, 16]. Though MSCs can be found in bone marrow, umbilical cord, fat tissue, and amniotic membrane, they are difficult to obtain for large-scale clinical applications owing to the limited sources and invasively collecting methods. HP-MSCs represent an emerging alternative source for tissue regeneration. HP-MSCs are advantageous over other MSCs for various reasons, such as their robust expansion ability, superior safety profile, and extensive sources. Noticeably, HP-MSCs can be directly applied to mouse models without immune response due to the low immunogenicity [43–45]. These advantages of HP-MSCs make them the ideal source for cell therapy. Interestingly, HP-MSCs are also found to improve angiogenesis, trigger regeneration of damaged organs [35]. These suggest that HP-MSCs based therapy is a potential therapeutic approach.

Mesh-like HA hydrogel can provide structural and mechanical support for HP-MSCs, and improve their retention time and therapeutic effects. Moreover, HA hydrogel is safe for mice and humans since it can be decomposed by hyaluronidase in the endometrium. In our study, HP-MSCs-HA could infiltrate and distribute both in functional layer and basal layer of endometrium, and retain longer than HP-MSCs. This effect is very likely due to the gradual release of HP-MSCs from the hydrogel, which indicated the great potential of HA hydrogel as an intrauterine controlled-release delivery system. Based on our study, 3D HA hydrogel facilitated more HP-MSCs survived and retained in the uterus to secrete more growth factors, which contributed to cell proliferation and angiogenesis within the endometrium. This paracrine effect may play a vital role in restoring thin endometrium, including the elevated endometrial thickness and embryo implantation rate. We further confirmed the effects of following treatments (HA group, HP-MSCs group, and HP-MSCs-HA group) on endometrial injury repair in the mouse model. There were no significant differences between the HA group and ethanol group in embryo implantation rate, gland number, endometrial thickness, and the IRS score of Ki67 and VEGF. Besides, no significant differences were observed between the HP-MSCs group and the ethanol group in increasing the number of glands and VEGF expression. Notably, HP-MSCs-HA treatment showed significance in all detected indexes compared with the ethanol group. Therefore, those results revealed the necessity of combining HP-MSCs with HA hydrogel in treatment of thin endometrium, which could help improve patient outcomes. Besides, Hooker et al*.* recently found that HA hydrogel therapy seems to improve fertility and reproductive outcomes in women, which indicated the extra beneficial characteristic of HA hydrogel on endometrial repair [10, 11]. Based on our study, HA combined with HP-MSCs therapy may be a promising treatment for patients with thin endometrium.

Cell proliferation, differentiation, angiogenesis, and migration are essential in re-epithelialization of the injured endometrium [46]. The ERK1/2 and JNK are two parallel MAPK signaling pathways in response to diverse extracellular stimuli, and they are involved in cell growth, differentiation, apoptosis, and inflammatory response effects [47, 48]. In our study, the protein levels of p-JNK, p-ERK1/2, p-STAT3, and VEGF were remarkably increased in HP-MSCs-treated endometrial stromal cells, which may partially explain the proliferation and migration-promoting effects of HP-MSCs on stromal cells. Consistent with our findings, evidence has shown that activating the JNK/Erk1/2-Stat3-VEGF pathway can promote the development of endometrial cells and likely enhance the uterine capacity [49, 50]. Jak2/Stat5 and c-Jun/c-Fos pathway play an important role in cell proliferation and migration. In our study, Jak2-Stat5 and c-Fos-VEGF pathways were upregulated in glandular cells when co-cultured with HP-MSCs, which may account for the increase of gland number in HP-MSCs treated group.

## Conclusions

In this study, HP-MSCs-HA were successfully prepared for the treatment of endometrial injury. HP-MSCs-HA exhibited a prolonged retention time in mouse uterus in contrast to HP-MSCs instillation alone. The in vivo therapeutic outcomes of endometrium-injured mouse models proved that HP-MSCs-HA could rescue the injured endometrium in mice via increasing the endometrial thickness and gland number, decreasing the fibrous area, promoting angiogenesis, and thus improving the implantation rate. The in vitro results suggested that HP-MSCs could promote the proliferation of human endometrial stromal cells by activating the JNK/Erk1/2-Stat3-VEGF pathway, and enhance the proliferation and migration of glandular cells via Jak2-Stat5 and c-Fos-VEGF pathway. Besides, HP-MSCs could also promote the angiogenesis of endothelial cells, which was consistent with previous in vivo results. Overall, our study provides theoretical and experimental foundations for the clinical treatment of thin endometrium using HP-MSCs-HA.

## Supplementary Information

**Additional file 1: Fig. S1**. The construction process of the endometrium-injured mouse model. I: mouse anesthesia; II: shaving the back of mouse; III: disinfecting exposed areas; IV: uteri exposure; V: instilling 25 μL ethanol in the uterine cavity and holding 3 min to fully establish the model of thin endometrium; VI: intrauterine instillation of 25 μL treating materials; VII: muscle suture; and VIII: closure of back skin incision. **Fig. S2**. Identification of human primary endometrial glandular cells and stromal cells by immunohistochemical staining of CK7 and Vimentin, respectively.

## Data Availability

The datasets used and/or analyzed during the current study are available from the corresponding author on reasonable request.

## Abbreviations

ART: Assisted reproductive technologies
BM-MSCs: Bone marrow-derived mesenchymal stem cells
FBS: Fetal bovine serum
FT-IR: Fourier transform infrared
GMA: Glycidyl methacrylate
HA: Hyaluronic acid
HA hydrogel: Hyaluronic acid hydrogel
HCG: Human chorionic gonadotropin
HP-MSCs: Human placenta-derived mesenchymal stem cells
HP-MSCs-HA: HP-MSCs encapsulated within HA hydrogels
HUVECs: Human umbilical vein endothelial cells
Jak2: Janus Kinase 2
MSCs: Mesenchymal stem cells
PBS: Phosphate buffer saline
SEM: Scanning electron microscopy
Stat3: Signal transducer and activator of transcription 3
Stat5: Signal transducer and activator of transcription 5
TEA: Triethylamine
UC-MSCs: Umbilical cord-derived MSCs
