# Supplementary material for: Synergistic regenerative therapy of thin endometrium by human placenta-derived mesenchymal stem cells encapsulated within hyaluronic acid hydrogels
Source: Stem Cell Res Ther. 2022 Feb 8;13:66. doi: 10.1186/s13287-022-02717-2 (PMC8822809; doi:10.1186/s13287-022-02717-2)
Supplement: Supplementary file 1 — Additional file 1: Fig. S1. The construction process of the endometrium-injured mouse model. I: mouse anesthesia; II: shaving the back of mouse; III: disinfecting exposed areas; IV: uteri exposure; V: instilling 25 μL ethanol in the uterine cavity and holding 3 min to fully establish the model of thin endometrium; VI: intrauterine instillation of 25 μL treating materials; VII: muscle suture; and VIII: closure of back skin incision. Fig. S2. Identification of human primary endometrial glandular cells and stromal cells by immunohistochemical staining of CK7 and Vimentin, respectively. [file 13287_2022_2717_MOESM1_ESM.docx]

**SUPPLEMENT MATERIAL**

Synergistic regenerative therapy of thin endometrium by human placenta-derived mesenchymal stem cells encapsulated within hyaluronic acid hydrogels

Yifeng Lin^1†^, Shunni Dong^2†^, Xiaohang Ye^1†^, Juan Liu ^1^, Jiaqun Li^3^, Yanye Zhang^3^, Mixue Tu^3^, Siwen Wang^3^, Yanyun Ying^3^, Ruixue Chen^3^, Feixia Wang^3^, Feida Ni^3^, Jianpen Chen^3^, Binyang Du^2^**^[[1]](#footnote-1)^***, Dan Zhang^1,3^*

^1^ Key Laboratory of Women's Reproductive Health of Zhejiang Province and Department of Reproductive Endocrinology, Women's Hospital, Zhejiang University School of Medicine, Hangzhou, Zhejiang 310006, China

^2^ MOE Key Laboratory of Macromolecular Synthesis and Functionalization, Department of Polymer Science & Engineering, Zhejiang University, Hangzhou 310027, China

^3^ Key Laboratory of Reproductive Genetics (Ministry of Education) and Department of Reproductive Endocrinology, Women's Hospital, Zhejiang University School of Medicine, Hangzhou, Zhejiang 310006, China


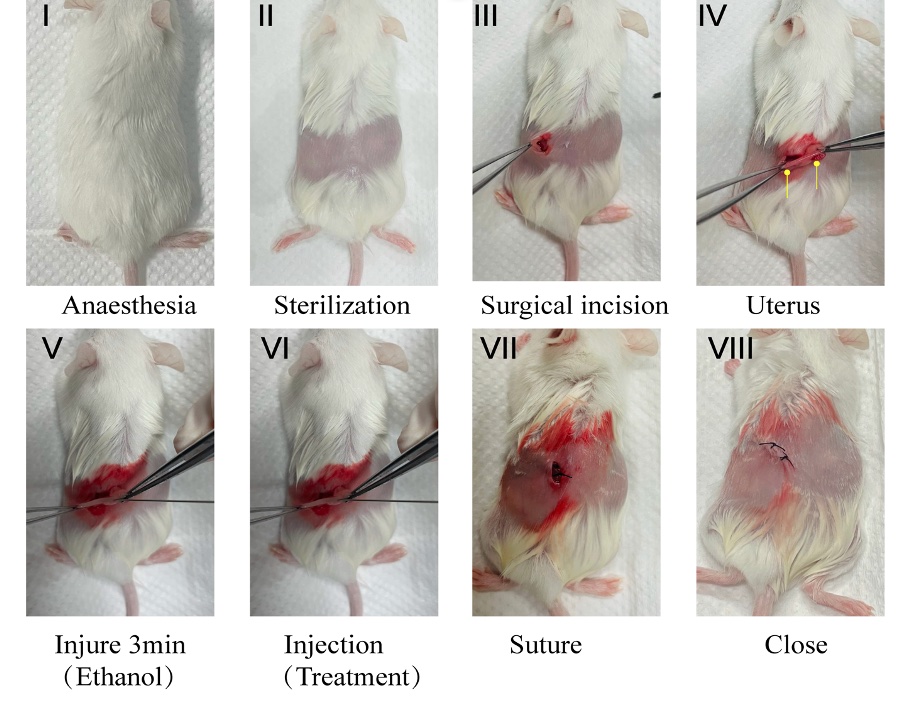


**Figure S1.** The construction process of the endometrium-injured mouse model. I: mouse anesthesia; II: shaving the back of mouse; III: disinfecting exposed areas; IV: uteri exposure; V: instilling 25 μL ethanol in the uterine cavity and holding 3 min to fully establish the model of thin endometrium; VI: intrauterine instillation of 25 μL treating materials; VII: muscle suture; and VIII: closure of back skin incision.

**
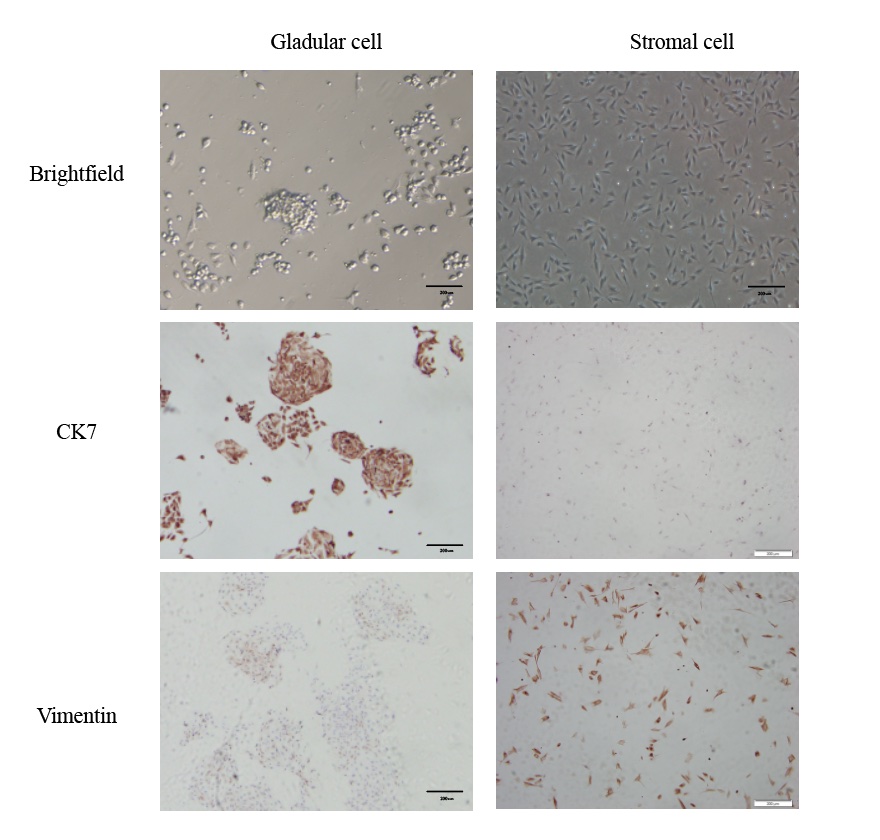
**

**Figure S2.** Identification of human primary endometrial gland cells and stromal cells by immunohistochemical staining of CK7 and Vimentin, respectively.

1. *Corresponding author. E-mail: [zhangdan@zju.edu.cn](mailto:zhangdan@zju.edu.cn); [duby@zju.edu.cn](mailto:duby@zju.edu.cn). [↑](#footnote-ref-1)
